# A simplified computational memory model from information processing

**DOI:** 10.1038/srep37470

**Published:** 2016-11-23

**Authors:** Lanhua Zhang, Dongsheng Zhang, Yuqin Deng, Xiaoqian Ding, Yan Wang, Yiyuan Tang, Baoliang Sun

**Affiliations:** 1 College of Information and Engineering, Taishan Medical University, Taian 271016, China; 2 College of Radiology, Taishan Medical University, Taian 271016, China; 3 Department of Sport Psychology, School of Kinesiology, Shanghai University of Sport, Shanghai 200438, China; 4 Centre for Psychological Health and Education, Dalian Nationalities University, Dalian 116600, China; 5 Interdisciplinary Center for Social and Behavioral Studies, Dongbei University of Finance and Economics, Dalian 116025, China; 6 Department of Psychological Sciences, Texas Tech University, TX 79409, USA; 7 Key Lab of cerebral microcirculation in Universities of Shandong, Taishan Medical University, Taian 271016, China; 8 Department of Neurology, Affiliated Hospital of Taishan Medical University, Taian 271000, China

## Abstract

This paper is intended to propose a computational model for memory from the view of information processing. The model, called simplified memory information retrieval network (SMIRN), is a bi-modular hierarchical functional memory network by abstracting memory function and simulating memory information processing. At first meta-memory is defined to express the neuron or brain cortices based on the biology and graph theories, and we develop an intra-modular network with the modeling algorithm by mapping the node and edge, and then the bi-modular network is delineated with intra-modular and inter-modular. At last a polynomial retrieval algorithm is introduced. In this paper we simulate the memory phenomena and functions of memorization and strengthening by information processing algorithms. The theoretical analysis and the simulation results show that the model is in accordance with the memory phenomena from information processing view.

Computer has excellent memory; we call it intelligent system working from input to output where the key hardware is memory. Humans can memorize; some of them have excellent memory, especially in short-term memory or instant memory. Mizraji^1^ ever said “Human brains are also information processing physical devices”. We assume that the memory of a person is the same as a computer; can we explain human memory by computer memory?

In order to verify this hypothesis, we present a simplified computational memory model from information processing view^2^; it can realize the memory and explain many memory phenomena although it is just a logic information processing model, not a biological model with biophysical or chemical signal processing. Our model handles the accurate and the context free data based on our research^3^, and the model is fit to semantic memory but not the context dependent semantic memory^1^.

As we all know, there are 10^11^ neurons and 10^4^ connections between a neuron with others^4^ in human brain taking part in the cognitive actions, so it is impossible to model memory from neuron grade thoroughly. Hence, researchers put forward all kinds of biological, physical and logical memory models with various methods; among them neural network has custom advantages of other fields^5^,^6^,^7^, but complex networks^7^,^8^,^9^ gradually achieved many results in recent years.

The objective of this study is intended to provide such a kind of modeling methodology for memory information processing; the model, called SMIRN, is represented by network with node and edge based on graph theory and complex networks^8^; we refer to the theories of hierarchical and modular architectures^5^,^6^ to directly develop the model from physiological and anatomical substrates, discarding the specific data surroundings and memory types. This work is a deepening of our small world memory network^2^ and is a meticulous model for memory phenomena with the accurate information processing. The representative memory models in these fields are summarized in section “Related Work”. We briefly summarize the theory and technology in section “Background” and have a detailed introduction to memory modeling and retrieval in section “Model”. Then we simulate the numerical memory algorithm and compare the retrieval efficiency to test the model in section “Simulation” and explain the memory phenomena with this model in section “Discussion”. At last we draw the conclusion of our simplified model and point out the shortage and the possible future research of our model.

## Related Work

In traditional memory studies memory has been accepted as network^10^, and visual modeling has been used from psychological to neural, physiological, anatomical, computational etc.^1^,^2^,^3^,^4^,^5^,^6^,^7^,^8^,^9^,^10^ around neuron, cortex, physical signal, chemical signal and information processing^9^,^10^,^11^,^12^,^13^; various memory networks were modeled from structural characters or functional characters such as analogy, dimensionality reduction^12^, classification^14^ and so on to study the associative^1^,^6^, free recall^15^, coding^16^, retrieval efficiency^13^ etc., and many different structures of the memory networks were achieved such as the modular^6^, the hierarchical^5^,^7^, the tree^17^ and the small world network^10^,^18^,^19^; the results simulated the structure, the function or the behavior partly or the whole, and some of them were hybrid.

Aimed at structural modeling, Renart and Rolls^6^ reported a multi-modular neural network model to simulate the associative memory properties from the neuron perspective in 1999; they used the tri-modular architecture to simulate multiple cortical modules and developed a bi-modular recurrent associative network from neuron and cortical levels for brain memory functions; in the network the local functional features were implemented by intra-modular connections, and the global modular information features were implemented by inter-modular connections. Ten years later, Rolls^9^ continued the computational theory of episodic memory formation based on the hippocampus and proposed the architecture of an auto-association or attractor neural network; in their results modular is a remarkable character. Meunier^20^ made a specialized study of modular for memory. Hierarchical^5^ is another remarkable character in memory network. Cartling^7^ had put forward a series of memory models and discussed the dynamics of the hierarchical associative process from the neuron; he emphasized the storage mode and information retrieval and mentioned the graph theory for memory network. Besides, Fiebig^21^ put forward a three-stage neural network model from autonomous reinstatement dynamics to discuss the memory consolidation.

Aimed at functional modeling, Lo^22^ used a mathematical model to simulate biological neural networks and proposed a functional temporal hierarchical probabilistic associative memory network. Lo simulated Hebb’s rule by a recurrent multilayer network and used a dendritic tree structure to simulate the neuron information input, moreover, he discussed multiple and/or hierarchical architecture. Polyn^15^ discussed the free recall process and reported the retrieval maintenance. Bahland^10^ delineated an efficient associative memory model by local connections and got a small-world network structure with high retrieval performance.

Of course, every structural or functional model is not independent, it is the integration of structural, functional and behavioral; the results fit for the computational modeling. Xie^12^ selected higher firing rate neuron to set up a low-dimensional network and discussed the functional connections by graph theory. Lu^19^ got a neuronal network of small-world from multi-electrode recordings related to the working memory tasks in the rat. Xu^23^ presented a simplified memory network aimed at pattern formation; they used two loops of coupled neuron to analyze the process from short-term to long-term memory theoretically.

Especially, memory simulating has attracted close attention aimed at information representation from structure to function, such as retrieval and efficiency. Just like Mizraji^1^ said, “Cognitive functions rely on the extensive use of information stored in the brain, and the searching for the relevant information for solving some problem is a very complex task”; they proposed a multi-modular network, which processed the context-dependent memory as the basic model; this model was developed according to the information query from brain dynamics searching perspective. Miyamoto^24^ reviewed the memory encoding and retrieval from the neuroimaging in primates. Tsukada^11^ used the real neuron as the basic structure to set up an associative memory model, which could realize successive retrieval. Bednar^13^ combined cortical structure and self-organizing functions to discuss the computing efficiency of memory network. Sacramento^17^ used a hierarchical structure to connect the neuron to improve the retrieval efficiency, and set up a tree associative memory network. Anishchenko^18^ pointed that the metric structure of synaptic connections was vital for network capacity to retrieve memories. Rendeiro^14^ improved memory efficiency by classification, but the model was hierarchical tree structure. Snaider^25^ set up an extended sparse distributed memory network using large word vectors as data structure, and got a high efficient auto-associative storage method using tree as data structure.

From the models above, we can find the memory networks have many remarkable structures and characters, such as modular, hierarchical, small world and so on^2^; with these characters the information processing can be high efficiency. It is ideal but difficult to model the memory thoroughly from the neuron level^9^,^11^,^24^. Flavell^4^ ever proposed meta-memory in 1971, and assumed that meta-memory was a special type of memory and represented the memory of memory, i.e., the reorganization, evaluation and monitor processes of memory in humanity itself. We introduced a logic conception of meta-memory^2^ to avoid the restriction of micro-scale. Meta-memory is an abstract definition, it includes a memory unit in the memory task, and it reflects the function of a neuron but not a neuron. Meta-memory represents an independent memory task unit or integrated information storage in our definition. Meta-memory node can be defined by different scales, for example, a meta-memory can be the memory of a number, a letter or a picture, thus, a neuron or cortices can be used as the meta-memory node in the memory network.

We ever put forward an initial memory model with small world characters based on the meta-memory^2^. In that model the cluster coefficient was discussed detailed but the algorithms were immature with ambiguous memory functions, in this paper we improve our retrieval algorithm and clarify the corresponding relation between the biological structure and information processing taking the word memory as example in order to refine our model in accordance with the memory functions precisely, such as the forgetting and association, which increases the map and understanding of the model.

## Background

This section introduces the theoretical background and key realization technology, which can help us understand the implementation and analysis of the SMIRN.

### Representation and algorithm

Many designers of memory model, like Tsukada^11^ and so on, think that the contribution of modeling for memory lies in the representation and algorithm. Representation in computer is the data structure, such as tree^17^ or vector^25^. In SMIRN, meta-memory is the basic unit, which expresses the information capability; it represents a flexible measurement, not a biological sense. In computer, the abstract data structure of MFset^26^ is similar in conceptual meaning, so we select set as meta-memory data structure and tree as the network structure. Set itself can store the information of meta-memory, and the functions of memory can be expressed by the results and performance of the information retrieval from set, so we need to introduce the retrieval algorithm of set.

Data structure and function are the basic components in an algorithm. MFset^26^ is a kind of set structure in computer, and its main functions include initial, find and merge. The set will be stored as a tree to finish the functions. Retrieval algorithms in MFset^26^ include find_mfset() (retrieving an element from set) and mix_mfset() (merging two sets). The find_mfset() is to find an element from the root to every leaf nodes in the tree. The mix_mfset() is to merge two tree, and the tree’s root of small connects itself as a new branch to the tree’s root of big number; the merge algorithm introduced path compression to reduce the depth of the tree, i.e., all the elements between root and finding element will be connected to root directly when the finding element is not on the second layer, named fix_mfset(). Time complexity is one of the important evaluation parameter in algorithm, i.e., the cost of time, which can be expressed by average search length (ASL). ASL is a mathematical expectation of the key times comparing to the given key, which indicates the mean number of comparison times in an algorithm, so it can be referred to the path length if the length of comparing key is used as a path in a network. For memory, the retrieval cost is the time complexity to find the context storing in memory network, which is used to evaluate the retrieval efficiency.

### Complex network and memory network

There is no doubt that the memory network is a complex network^8^, which has attracted much attention in real complex systems since Watts^27^ proposed the small-world network model in *Nature*. Complex network has been one of the hotspot tools in science and nature based on statistical physics, graph theory, control dynamics^28^ etc. Meanwhile, the rapid developments and applications on memory with complex network have brought us a lot of results ranging from microscopic to macroscopic, also from structural to functional^10^,^12^,^18^,^19^.

In general, the objective of a real system with complex network is the modeling and the application; both of them follow the modeling process of complex network including the definitions of node and edge, the evolution of network, and the computation of topology properties. For memory network, the functions need to be calculated by the topological properties to investigate biological memory. Complex network has three basic properties, degree distribution, average path length (APL), and clustering coefficient^8^,^27^. Degree distribution is the distribution of the degree of each node in the network. APL is the mean length of the network; the mean is the shortest distance between any two arbitrary nodes in the network, and the distance is the geodesic distance, not the Euclidean distance, indicating the edge number of the shortest distance between two nodes. Clustering coefficient is the mean of all the nodes in the network, which represents the degree of importance of nodes and the group degree in the network, i.e., the modularity degree, which is higher if the clustering coefficient is higher. Besides, complex network has the properties of modularity, centrality, robustness, cost, efficiency and so on; all the properties can be gotten by the three basic characters. Topological properties stand for different meanings for different networks. For the memory network, the degree distribution stands for the opportunity to participate in processing information of meta-memory; the APL stands for the efficiency of processing information, that is the cost of retrieval meta-memory; the clustering coefficient stands for the whole efficiency of processing information of memory.

It is well know that memory can be expressed by functional networks^19^,^23^. With complex networks we can compute the memory function from qualitative to quantitative.

## Model

In this section, we describe and explain memory functions and information processing by the word memory^16^. We know that the memory starts from studying or training; it is the beginning of the memory information processing. Memory includes three operations, memorization, strengthening and forgetting from information processing, and the results of three operations can be represented as cognitive functions such as association. We get a bi-modular hierarchical memory network by a modeling algorithm and a strengthened memory network by a polynomial retrieval algorithm.

Memorization is the process that a more consolidation temporary contact forms through the sensory perception, such as hearing or vision, and the memory information will be stored in the brain by memorization. Strengthening is the process that the contact becomes more closely if the same context is repeated perceived. Forgetting is the process that the contact becomes weaker and weaker until vanishing if the same context is no longer perceived with long time from part to whole. The strengthening or the forgetting will happen when we successful retrieve the memory after the memorization. In accordance with the memory operations from computer, the memorization is the node addition of set element to the tree network, the strengthening is the edge updating and the forgetting is the edge deletion from the tree network, of course the node. The functions of association and recall contextual are weak in this model which can associate limited content or simple answer for recall. To model the memory network, the node and the edge of network will be defined based on neurobiology. Node is an independent unit as the basic component in network. Edge is the relation of nodes to form network by linking nodes. Path is a collection of edges and nodes connected.

We assume several functions on neurons (nodes) in order to demonstrate word memory.

1. A node can store a letter.
2. A node can compare a stored letter with a letter sent from the parent node.
3. A node can respond differently depending on the result of (2).

(3–1) When the sent letter is the same as the stored letter, the rest of letters are sent to the next node.

(3–2) When the sent letter is different from the stored letter, the node can send failure signal back to the parent node.

Memorization is to set up network in memory network. A node is a meta-memory unit, perhaps a number, a letter, or a picture. Interrelated nodes are the nodes in a memory task, perhaps a sentence, a word, or a piece of music melody; the interrelated nodes form a path. For example, the letter “n”, “e”, “t”, “w”, “o”, “r” and “k” are a node respectively and they connect to a path when we learn the work “network”. Similarly, we need to memorize the single tone and melody when we learn a piece of music; a single tone is a node, and a melody is a path. The memory network or the music network forms when nodes and edges are stored. We introduce the modeling algorithm to describe the whole processing and the memory network SMIRN can be gotten with the modeling algorithm. Strengthening is a continuous searching process for information processing. We can confirm the memory in our brain by searching and display the memory content from our brain quickly, and we introduce the retrieval algorithm to express the strengthening to demonstrate the process. Retrieval in the model is to look for the content, and the operation to all the behaviors is retrieval. The retrieval requirement includes recall and recognition. Forgetting doesn’t need the brain to take part in the process actively and doesn’t need algorithm to achieve, which is just a deletion operation from SMIRN.

To be accordance with the computer memory, we set the root node as the input and output positions, that is the beginning and end positions of memory. The beginning stands for the retrieval requirement in our model. The end stands for the failure of remembering if the output is null and the success of remembering if the output is not null. The output is different from retrieval requirement. We can output the yes/no or exist/not exist for recognition, also we can output the finding tree if the requirement is write down or recite the spelling of the word, but the process should add the output of the finding tree after success retrieval. It should be noted that this model is not fit for recall which need us to expand with edge weight in the future based on SMIRN. The path between root and stored node (related to the old) is shorter with the old or related content when strengthening happens, and those nodes’ paths that lie between root and finding nodes also become shorter. Otherwise, the path will be longer after a long time with no studying when forgetting happens.

### Bi-modular hierarchical memory network

We simulate memory from the information process perspective referred to the anatomical structure. In anatomy, the structural unit includes the neuron and the brain region. When the memory task comes, the neurons take part in first and every neuron has itself memory task, the information begin to store in neuron, and a node forms in network. Then the neurons integrate together by some type, and part or whole information are stored; the edges form to link the neuron nodes in network, more and more nodes and edges form the inner memory network. A cortical node forms or the nodes and edges of outer form just like the neuron nodes and edges if the neurons in a single brain cortical region and the difference of the two kinds of nodes and edges are intra-modular and inter-modular.

Function, information and network are three different types to describe memory in the whole life cycle of memory from beginning to end. We focus on memory functions to simulate the forming process and model the evolution of memory. Memorization, strengthening and forgetting in memory reflect on information processes are storing, updating and deleting, and reflect on networks are adding or deleting nodes and edges, shortening or lengthening path length. For example, given a memory task “complex network”, the two word information are stored in brain when we memorize, and the storage form in network are two paths “c-o-m-p-l-e-x” and “n-e-t-w-o-r-k”, and then link the two paths to form the network with a root. Every letter is a node in the network, and every “-” represents an edge to link the nodes. The letters in a word are interrelated nodes. From “c” to “x” is a path. The “n-e-t” and the “w-o-r-k” also will be strengthened if we learn “network” repeated, and the path will be shorter. Meanwhile, the word “netbeans” also will be strengthened and the retrieval path will be shorter because of the shorter path of “n-e-t”. Except that, we make some hypotheses and simplifications, then we develop a hierarchical model that is composed of the inter-modular and intra-modular models, i.e., a bi-modular model. An intra-connection is established to reflect the neuron and synaptic, and an inter-modular connection is established to reflect the brain cortical by introducing meta-memory. The bi-modular model is set up separately.

It is unknown in precise image for neurons to process information^1^ and different for neurons connection between anatomical structure and information processing^12^. We know that not all the neurons take part in a cognitive action from the Hebb rule, even the adjacent neurons^11^,^22^. Xie^12^ just selected the higher firing rates neuron to model, which also provides support to us for our modeling methodology that is our focus on the information processing, not the true structure and sequence of neuron. It is allowed for us to close the anatomical structure on our information processing structure in case of no violation of biological structure to better understand information processing of our model. Meanwhile, the emphasis of our model is information processing for memory, not the biological neural network. The aim is to improve similarity and mapping in appearance.

A neuron is looked on as a node from the structural for the intra-modular model to be accordance with the general image of synaptic, and the information transmitter is regarded as the edge between the neurons^6^,^9^,^12^,^19^, such as the excitatory or inhibitory neurotransmitters. The node and the edge connecting together look like the neurons connecting in neurobiology structure. All the neurons taking part in form a network modular for a given task, i.e., the intra-modular. Then, a certain brain cortical region is looked on as an inter-modular node when the neurons taking part in the task belong to different brain cortex, such as the hippocampus or prefrontal cortex; the different cortical regions connect each other in anatomical structure^1^,^7^, and the edges form if two cortical regions are adjacent in anatomical structure. The inter-modular node is the super node that represents the entrance of the memory storing and retrieving, meanwhile, it is the root of the intra-modular model in the memory network^24^. The inter-modular forms the outer network and the intra-modular forms the inner network, thus the hierarchical structure form. The intra-modular can transport information by inter-modular.

### Modeling

We can model it by intra-modular and inter-modular respectively by the description of bi-modular memory network. The memory actions are not isolated in intra-modular with a single neuron and the triggered neurons connect together and form the intra-modular network by memory actions; it should be regular and sequential in the formation processing. The memory begins to form when we learn new knowledge or execute new cognitive action, and the memory information begins to be processed and stored in our brain. We use the modeling algorithm to simulate the process to memory the word “netbeans” and “network”, and we can get the results as below by the modeling algorithm.

**Figure 1:**
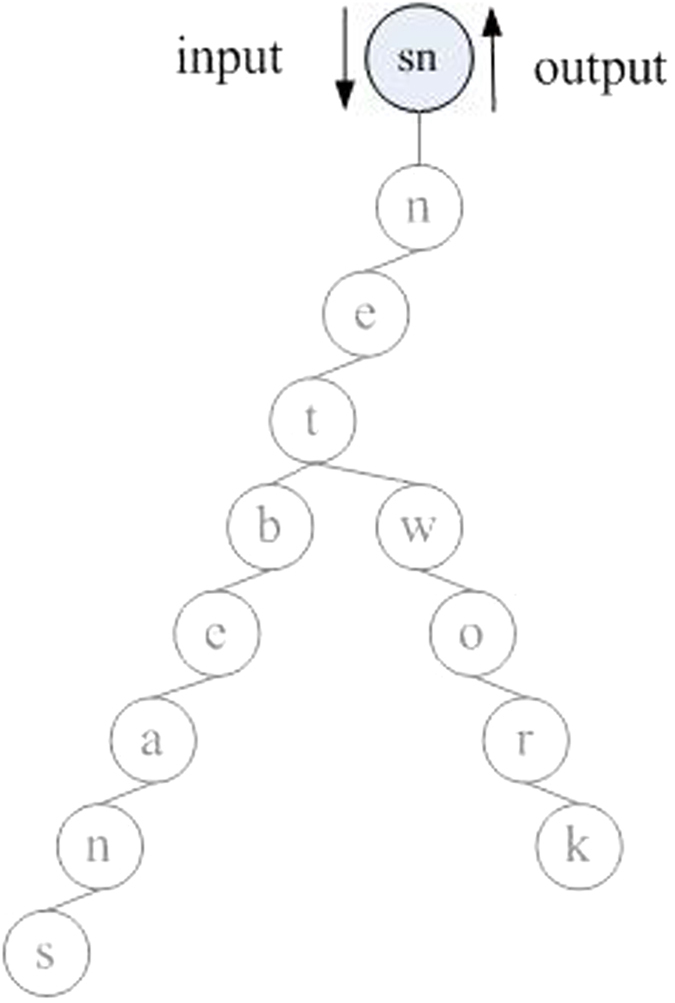
The intra-modular tree model in SMIRN with words memory of “netbeans” and “network”. The information is inputted into the superior node (sn) with the neuron node and the edge connection; paths form the intra-modular model; the output expresses the retrieval operations.

Clearly, the intra-modular model is finally constructed as an inverted tree^17^,^25^ (Fig. 1). The tree is the memorization form. The strengthening process will be realized by retrieval algorithm subsequently. Forgetting becomes when we can’t recall the content ever in our brain which is different from misremembering, and this process doesn’t need the neuron or cortex to participate in information processing because it is a passive process. Forgetting includes the partial forgetting and whole forgetting. We define a class of virtual node which has no effect on the construction process of SMIRN to describe forgetting function. The virtual node is tip node and end node from computer definition, which can enter the network with time, of course, it doesn’t exist in the anatomical structure of the brain represents the forgetting function in the memory information process. A virtual node joins the network similar to the addition of a new node to the network; the difference is that the virtual node connects the network from the lowest position, i.e., treetop, climbing to the upper step by step. At first, it replaces the last node and contacts the network; the partial forgetting begins to happen. Then, it replaces its parent node until to root; the whole forgetting comes. Of course, the virtual node hasn’t child node when it connects to the network or climbs to the upper, and the replaced node will be deleted. A virtual node along with its edge disappearing from the network is the the process of deleting, which is simpler than the strengthening process.

An incoming memory tasks develop a memory network, including forming a new network or strengthening the old network with the modeling algorithm (Methods). The new network is the step (2) “network growth” of (a) “a new node”; the strengthened network is the step (2) “network growth” of (b) “an old node or path”. Besides, weakening forgetting can also strengthen network by removing the virtual nodes gradually.

**Figure 2:**
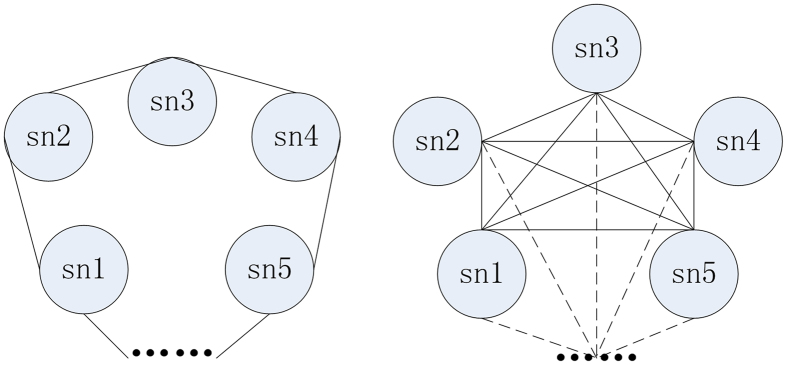
The extreme connections in the inter-modular structure of SMIRN; the brain cortical regions as the super-node forms a connected ring (left) or a full connected network (right).

For inter-modular, it is well-known that many cortex take part in the memory process^6^,^7^, therefore, we design the inter-modular structure to describe the anatomical and functional connections of brain based on the intra-modular structure. Theoretically, a connected ring and a full connected network are the extremes to connect a network for the inter-modular structure (Fig. 2). According to previous studies^6^,^10^,^18^,^19^, we know that the network of inter-modular is not great because not all the brain cortical take part in the memory function. We conclude that the largest isn’t more than 90from anatomical automatic labeling (AAL) model with the brain dividing into 90 cortex^29^.

Combining the inter-modular and the intra-modular structures, a bi-modular hierarchical network is formed, and the brain stores memory information by SMIRN. For the implementation, we use parent–child-linked list as data store structure, which has been defined as the child–brother data structure in definition of MFset^26^. For example, the hippocampus or prefrontal cortex can be looked on as the inter-modular which can be implemented in the computer by a cluster of linked path if we memory a paragraph, and every word can be looked on as the intra-modular which can be implemented in the computer by a linked path.

### Polynomial retrieve

When we learn old knowledge or execute ever cognitive action, it is no longer the process of memorization, but a more clearly memorization process based on the initial memorization, i.e., strengthening process. How do we know that we have remembered the content? It can be by searching and confirming processes. The memory will be more clear and faster if we remembered. We introduce a retrieval algorithm to describe the processes and results.

It is well know from the anatomy that so many neurons take part in any brain cognitive function. Comparing with intra-modular, the biggest length (ASL) of the retrieval path for inter-modular structure is 90 based on AAL model^29^ when the inter-modular is a connected ring, and it is 2 when the inter-modular is a full connected network, moreover not all the cortical areas participate in the memory process^10^. We can set inter-modular aside temporarily and emphasize on the data querying from the intra-modular structure. Therefore, our retrieval algorithm is an intra-modular retrieval algorithm.

Compared with our previous model^2^, we improve our retrieval algorithm so that it can be in accordance with the memory function phenomenon from the biological view. For example, all the retrieved contents should be more closely in the inter-modular node, and can’t be free combination and association. The retrieve process is as below by the retrieval algorithm.

In the retrieval algorithm, it will be a successful search process if the incoming memory event exists, otherwise, the search fails if the incoming memory event does not exist, and a new memory network is developed by modeling algorithm. The process is just like the existing incoming memory for the existing memory events, otherwise, it is forgetting; if it is a partial forgetting, the part successful search will be strengthened and the part of forgetting will be deleted from the network directly, i.e., discarding of nodes and edges; a virtual node will be added in the last position. If it is forgotten completely and can’t be remembered, the whole path will be deleted from the network, meanwhile, a new memory network will be reconstructed by modeling algorithm. For the simulation and implementation of the retrieval algorithm, we can refer to the functions of fix_mfset() and mix_mfset() in the MFset^26^, including the node-finding and edge-changing operations.

Based on retrieval, we can calculate the memory efficiency by retrieval time and path length, i.e., cost. The efficiency is referred to the retrieval efficiency in SMIRN, i.e., the efficiency of strengthened SMIRN, meanwhile, cost is referred to the entire output cost; we define cost(*n*) as the intra-modular cost and cost(*sn*) as the inter-modular cost, then the memory retrieval cost of the SMIRN is the sum of cost(*n*) and cost(*sn*).

## Simulation

Our model is a kind of information processing memory model, which is specialized at precise memory and search, and it doesn’t fit for the semantic and episodic memory. The processes of modeling and retrieval are objected to precise content, which don’t fit for the memory type of storage and association from contextual and semantic. With our model, it can store all the information that can be expressed by the deterministic data structure and remember the content with time depending on familiarity, of course, it can forget the content without studying for a long time.

We make SMIRN memory the new words with a paragraph by the modeling algorithm in order to show the image of our model. After that, we verify the retrieval algorithm efficiency by retrieval algorithm. In word memorization, a new word as a path will be added to the model, and the repeating words or the interrelated words can strengthen the network and the path will be shorter. In the simulation, we select the ABSTRACT paragraph as memory tasks of the paper refs 2,7,8. We suppose that only one cortical takes part in the model and all the neurons connect to the cortical. The neuron and the cortical all can be looked on as meta-memory, and they are different in the meaning but no effect on the path computation from information processing view. In the figure, the center node is the root node (sn) where the memory information is imputed in or outputted from the network. Every node stands for a letter and every branch stands for a word, the different branches beginning from the root to the treetop stand for different words. The SMIRN of all the words in ABSTRACT paragraph of Ref. 7 is Fig. 4 by modeling algorithm^2^.

We set four brain cortical regions to simulate the bi-modular structure with the connected ring to strengthen the comparison; the four brain cortical regions memory four groups of word (a-e, f-l, m-q, r-z) respectively in Fig. 5.

We didn’t simulate the forgetting process in the simulations, so there is no virtual node. The virtual node will be added to different branches and the corresponding path will be shorter if the forgetting happens. We compare to the MFset finding algorithm with the same ABSTRACT paragraph of ref. 7 to show the high efficiency of our retrieval algorithm. The memory efficiency depends on the cost, i.e, the search length in memory network when we retrieve the memory network. In our model, the result is thoroughly the same comparing the definitions of APL and ASL if we compute the APL of the root, not the whole network. From the definition and derivation of APL, it can be concluded that the average of the network’s APL is on the same degree of magnitude. From the definition and derivation of ASL, the similar conclusion can be concluded. So we can use the value of ASL to represent the APL. Because the path length stands for the network efficiency, we calculate the ASL of the MFset finding algorithm and our retrieval algorithm, and the results are demonstrated in Fig. 6. Line SMIRN(1) is the result of Fig. 4; line SMIRN(1b) is the result of Fig. 4 with an inter-modular node so that it is a bi-modular network; line SMIRN(4) is the result of Fig. 5.

Figure 6 shows the comparison of SMIRN retrieval algorithm with the MFset retrieval algorithm and the result indicates the effect of the retrieval algorithm has the similar degree of ASL with the MFset. Because of the polynomial time complexity with fix_mfset() of MFset^26^, we can conclude the polynomial time complexity of our retrieval algorithm. Even it is not so good as MFset which is ideal for memory, it is still effective. The ASL of SMIRN retrieval algorithm is convergent to 2, which indicates that the cost of the model is small. Moreover, it supports that the bi-modular memory network model has a small-world character. Besides, compared with our previous model in retrieval algorithm^2^, we find the big difference. In Fig. 5, MFset is smaller than SMIRN especially in the beginning which is completely opposite conclusion with our previous model^2^ that is an ideal state, so we think it is more persuasive to explain memory process.

## Discussion

**Figure 3:**
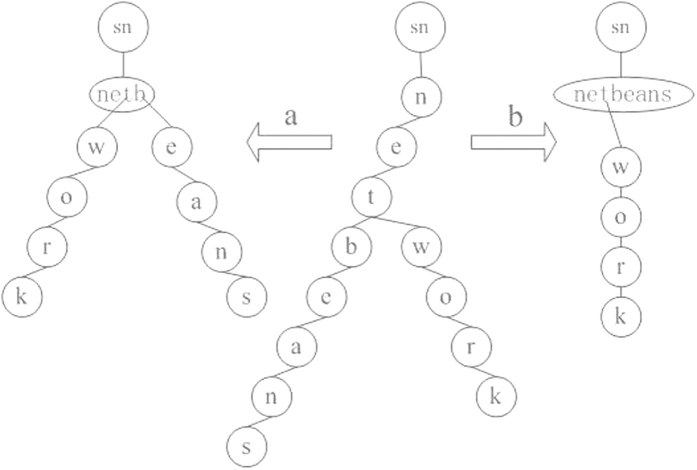
Illustration of the retrieval of a node or a path. If a single node (the letter “b”) is retrieved in intra-modular tree (**a**), it connects to the root (sn) directly with its neighboring nodes (the letters “t”, “e” and “n”), and the edges are discarded with their parent nodes. Simultaneously, all other nodes also change the path, undoubtedly becoming shorter to the root. If a path (the word “netbeans”) is retrieved (**b**), they and their neighboring nodes connect to the root directly, and the edges are discarded with their parents.

**Figure 4:**
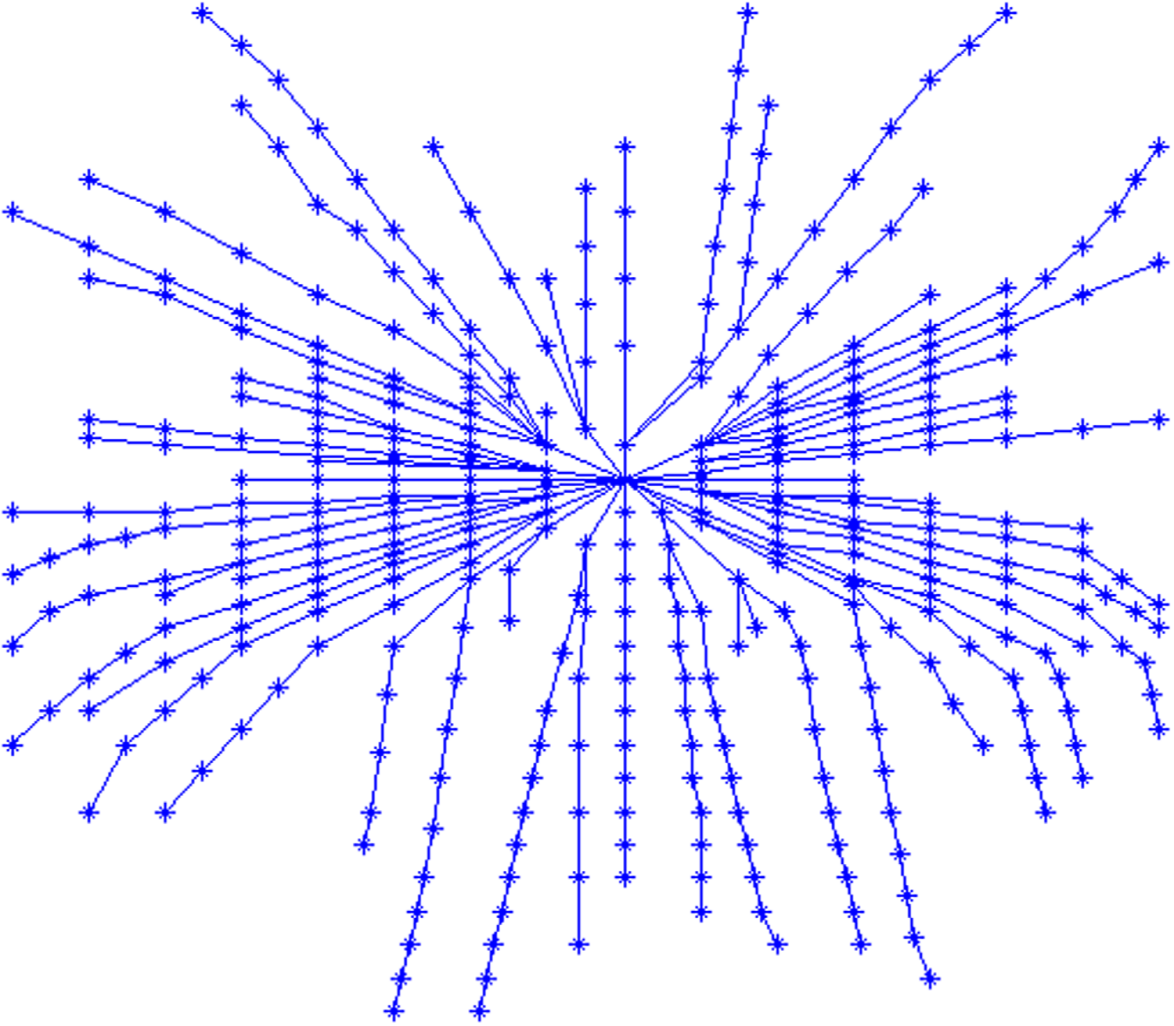
Simulation of memory network model by modeling algorithm. The output of the model is the root of the intra-modular structure in the network regardless of the inter-modular structure.

**Figure 5:**
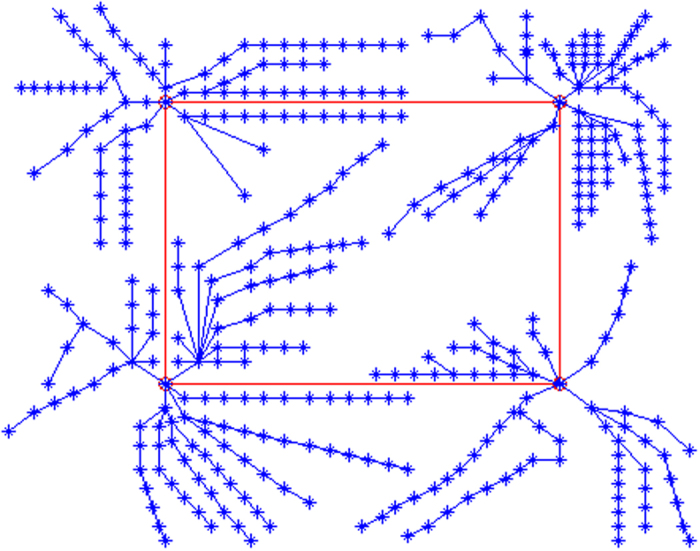
Simulation of memory network by modeling algorithm with four brain cortical regions.

**Figure 6:**
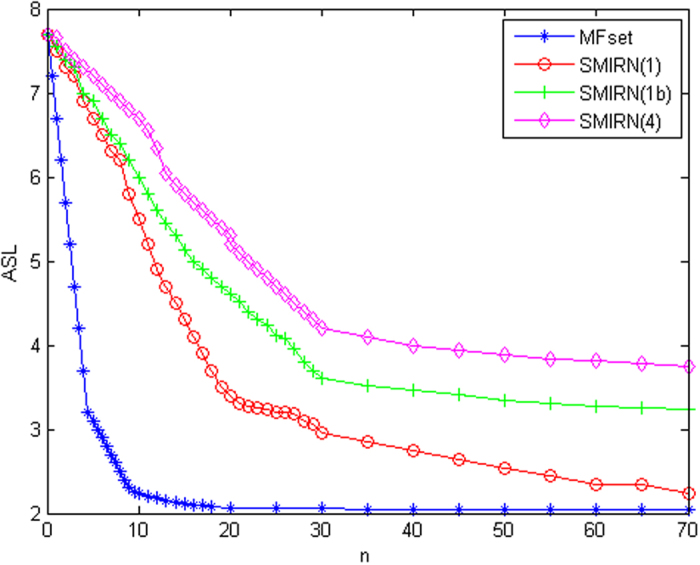
ASL in SMIRN retrieval algorithm and MFset algorithm (fix_mfset()) with increasing retrieval step *n*.

We can explain the memory functions from the results of our model and algorithms to verify our model and algorithms being accordance with memory functions. In this paper, a meticulous SMIRN model a retrieval algorithm with polynomial time complexity were described based on our previous model^2^. The function process of memory was simulated from the information processing. The SMIRN is a deepening and more meticulous model compared with our previous model^2^; the methods of modeling algorithm and retrieval algorithm are similar because of the same process from initial state to growth or retrieval. The differences are also obvious; first we introduce the forgetting in SMIRN which makes the memory more reasonable and perfect, and with forgetting the memory becomes a complete cognitive activity; second is the data structure in retrieval algorithm and the process in Fig. 3 is more reasonable, i.e., all the nodes on the path directly connect to root is not so reasonable and a little difficult whether from the complements or from memory phenomena.

In the beginning of Introduction, we said that the computer and human had excellent memory quality. The excellence can be calculated by the cost of the retrieval. From the retrieval algorithm, we know when we repeat to study the words, more and more nodes will be connected to the root, and the path will be shorter and shorter. At last to the extreme, all the nodes will be connected to the root, the memory effect is best; of course the forgetting will also come with time. The retrieval cost depends on the time complexity and the space complexity from computer. In the MFset definition^26^, the time complexities of fix_mfset() and mix_mfset() algorithms were proven to be *O*(*nα*(*n*)); *α*(*n*) is the functional inverse of Ackermann’s function, whose growth rate is much slower than that of function log*n*, for example, when *n* is 2^21019729^, *α*(*n*) = 4^26^, so for the common *n*, *O*(*nα*(*n*)) is the polynomial time complexity. From the realization of SMIRN and MFset, a trivial difference was observed in the performance of the retrieval algorithm comparing to the fix_mfset() and mix_mfset() functions. For the space complexity, compared to the parent-linked list^26^, a parent-child-linked list will cost slightly more, but it is acceptable (or negligible) for faster development on computer storage capacity. For the time complexity, the node-finding operation requires to search child node(s) except the parent node on the implement of fix_mfset(), but the increment is a variation of the coefficient for the polynomial parameter, i.e., *cn*, not of exponential parameter. The edge operation has no difference with the mix_mfset() function because the change from the initial state to the final state is identical, and the final state of edge change is only one of the middle states of the mix_mfset(); therefore, the cost(*n*) is *nα*(*n*)+*cn*, and it is the same grade as *O*(*nα*(*n*)), i.e., polynomial time complexity. For the cost(*sn*), the APL can’t exceed 90 based on AAL model^29^ whether the connection is a full connected network or a connected ring, so the time complexity is also polynomial.

As we know, the APL can be computed by the cost and the cost of the SMIRN is the sum of inter-modular and intra-modular structures. The APL of SMIRN can be regarded as the sum of path lengths with value one and value two; value one is between root and internal node, and value two is between the internal nodes; clearly, value one is 1 and value two is 2, so the bi-modular network has a small APL which represents the high efficiency. The clustering coefficient is the number of node *sni*, i.e., the number of brain cortices taking part in the memory process, it is clearly 1 because of the inter-modular node’s selection in the simulation, and the number depends on the inter-modular structure which is high because of many brain cortices taking part in the memory functions. Moreover, the node in intra-modular structure clusters to the root with the retrieval algorithm, and finally form the inter-modular clusters with the high cluster structure. Based on the above findings, the SMIRN is determined to be one of the small-world networks^2^.

In memory cognitive functions, forgetting is a common phenomenon and it corresponds to the cases when no same or similar memory contents are input into the memory for a long time. Based on the definition of virtual node, the forgetting process involves the gradual removal of a neuron node or path from the root with the insertion of virtual node. Here, we can introduce a time threshold^9^ to decide to delete a node; the virtual node replaces the node if the time exceeds the threshold. The forgetting process has no effect on retrieval algorithm, because a virtual node represents a node only. We can use strengthening to explain the association function to a certain degree, for example, the letters “n”, “e” and “t” will be associated when we retrieve the letter “b” in Fig. 3; the words “net” and “bean” will be associated when we retrieve the work “netbeans”.

It is well known that the memory is a kind of cognitive process, and the results of the cognitive are the information memorized or forgotten. The information can be expressed by the memory networks^19^,^23^. The memory network has the advantage to express the cognitive actions just like the application of other networks, such as Boolean network, the directed network, etc. Our information retrieval network can express the memory process and explain the memory phenomena, and it provides a new explanation for memory cognitive actions from information processing, moreover, we can quantitatively discuss memory function from information processing to some extent, so our model is consistent with the conclusions of lots of existing models from the results. Our model is modular and hierarchical from the structure which is the same as multi-modular memory model^1^,^6^,^9^. Renart^6^ put their emphasis on the knowledge input and out express; our model puts emphasis on the information storage and retrieval, which is similar as Mizraji^1^. Fiebig^21^ and Fujita^16^ put forward the hierarchical model and put emphasis on knowledge express or anatomical structure. For the meta-memory, Tsukada^11^ used the real neuron to simulate the memory and Flavell^4^ also used the memory-meta to deduce the complexity, but our model introduces the meta-memory to solve the logic representation of neuron and cortex. Our memory network is a small world network from the functions which is the same with the results^10^,^18^,^19^, but different from Xu’s model^23^, which had scale free characters. We get an inverted tree as the results^17^ from the network structure which expresses the similar hierarchical structure and high retrieval efficiency. Especially, if our model memorizes the default content, such as a misspelled word, the misspelled word is also constructed in the network by a different path, as long as we don’t repeat the misspelled word, but it will be gradually forgotten for a long time. The misspelled word may be strengthened part of the word when we retrieve, but it is impossible to be outputted except that the memory requirement is the misspelled word. Moreover, if there is no right spelled word corresponding to the misspelled word memorized in the model, the right memory requirement is failure, i.e., the output is null.

## Conclusions

In this paper, we propose a simplified computational memory model with bi-modular hierarchical structure from the information processing view based on complex networks and computer algorithms including memory formation and retrieval. The objective of our paper is to set up information processing memory model to simulate the phenomena of memorization, high efficiency instant memory, forgetting and so on, so that our model can meet the characteristics part of memory.

It is well known that memory is a type of advanced neurocognitive action and transfers information by the interactions of neurons from anatomical perspective, and it can be considered as the memory functional connections of the neurons and cortices from the brain functional characters. Memorization is the selection and perception to things and the main process of information encoding from information process. Strengthening is a neurocognitive process for the past things and a dynamic process in the brain.

This paper is intended to provide a simplified computational model, perhaps it is a simple cognitive model, but it simulated the memory processes by a computational model from the beginning to the end. SMIRN simulates the memory structure and function in an acceptable way although it has not established biological verifications, and it can only be defined to a functional network model rather than a structural network before the biological verifications are achieved. Meanwhile, our model is not a thorough and strict simulation for all memory structure. A thorough and strict memory model should include more inter-modular and more complex tasks so that it is more like a complex network, but it is consistent with the complex network from the discussion and results. We developed a small-world SMIRN successfully by abstracting functional connection based on the graph theory from complex network. The physiological structure is not so strict, but produces a new hierarchical bi-modular model from information processing, whose advantages can not only explain the functions with bi-modular, but also can express the structure of neuron and cortex with hierarchy. The model is in accordance with other hierarchical and/or modular memory networks^1^,^6^,^9^,^16^,^20^,^21^ and small-world networks^10^,^18^,^19^.

Of course, several aspects of this model should be improved^6^. The edge connection effectively reflecting the cost of the network doesn’t consider the weight which can better reflect the network characters qualitatively, not quantitatively. The value of forgetting threshold ought to be more systematically and scientifically defined, measured, and confirmed under the theoretical and experimental conditions. More research should be put on association mechanism and degree. However, SMIRN is impossible to explain all the biological structures and cognitive phenomena appearing in neural networks and memory models even if we improve all the components. SMIRN has made progress comparing with our ever model^2^ in the explanation of formation, process, structure and functions of memory. Meanwhile every attempt to uncover the mystery of memory implies that SMIRN is worth pursuing further as a functional model.

## Methods

### Modeling algorithm

A path forms when the nodes are connected by the edges and the information in one path represents consistent or correlative response. We assume that all the information is processed via the root, i.e., storing or retrieving the memory with input or output operations via the root^9^. Moreover, for simplicity, only the active neurons in memory can be considered from the function, not from anatomical structure, even if they are adjacent; meanwhile all the neurons are assumed to be in the same state^1^. We provide a regular and sequential process to form the intra-modular network as follows^2^:

1. Initial condition: Beginning with the root node and waiting for memory input, i.e., memory requirement.
2. Network growth: The nodes are added to the network or strengthened selectively when the memory inputs, i.e., word comes. If the input is

- A new node: It is added to the network directly and connects the root by an edge automatically. In particular, if the input is a series interrelated nodes, they are added to the network in the form of a path, which is connected sequentially before adding to the network, and the process is similar to the new node.
- An old node or path: i.e., the same or similar memory contents, they are strengthened in the network by the retrieval algorithm.

### Retrieval algorithm

The retrieval is an output process including the data querying from the intra-modular structure and information integration among the inter-modular structures corresponding to the bi-modular hierarchical structure^6^,^10^. The result is a node if the retrieval is an independent result, such as the letter “a”. The result is a path if the retrieval is a word, such as “network”. The retrieval algorithm of intra-modular structure can be described as follows to simulate memory strengthening function and information retrieval process^2^.

1. Initial condition: Beginning with the intra-modular structure and waiting for memory retrieval, of course, a bi-modular hierarchical SMIRN has been constructed.
2. Retrieval output: The retrieval process occurs on the premise that the retrieval data exist in the intra-modular structure when the memory is required. The neighboring nodes including the parent node and child node(s) are defined, not the brother node(s), and the data is queried from the root in the inverted tree. All the retrieved nodes and their parents (if existing) connect to the root node directly if the result is a node or a path and their former relations to their parents of the retrieved nodes are discarded, but their former relations to their children remain unchanged, and the path length to the root of their children becomes shorter. For example, the retrieved node is connected directly to the root along with its parent nodes except the node that has been connected to the root originally (Fig. 3a) if we retrieve a letter, and it is similarly if we retrieve a word more than a letter, see Fig. 3b.

## Additional Information

**How to cite this article**: Zhang, L. *et al*. A simplified computational memory model from information processing. *Sci. Rep.* **6**, 37470; doi: 10.1038/srep37470 (2016).

**Publisher's note:** Springer Nature remains neutral with regard to jurisdictional claims in published maps and institutional affiliations.
